# Co-infections and transmission networks of HCV, HIV-1 and HPgV among people who inject drugs

**DOI:** 10.1038/srep15198

**Published:** 2015-10-13

**Authors:** Kim Tien Ng, Yutaka Takebe, Jack Bee Chook, Wei Zhen Chow, Kok Gan Chan, Haider Abdulrazzaq Abed Al-Darraji, Adeeba Kamarulzaman, Kok Keng Tee

**Affiliations:** 1 Centre of Excellence for Research in AIDS (CERiA), Department of Medicine, Faculty of Medicine, University of Malaya, Kuala Lumpur, Malaysia; 2 AIDS Research Center, National Institute of Infectious Diseases, Toyama, Shinjuku-ku, Tokyo, Japan; 3 School of Medicine, Yokohama City University, Yokohama, Kanagawa, Japan; 4 Division of Genetics and Molecular Biology, Institute of Biological Sciences, Faculty of Science, University of Malaya, Kuala Lumpur, Malaysia

## Abstract

Co-infections with human immunodeficiency virus type 1 (HIV-1) and human pegivirus (HPgV) are common in hepatitis C virus (HCV)-infected individuals. However, analysis on the evolutionary dynamics and transmission network profiles of these viruses among individuals with multiple infections remains limited. A total of 228 injecting drug users (IDUs), either HCV- and/or HIV-1-infected, were recruited in Kuala Lumpur, Malaysia. HCV, HIV-1 and HPgV genes were sequenced, with epidemic growth rates assessed by the Bayesian coalescent method. Based on the sequence data, mono-, dual- and triple-infection were detected in 38.8%, 40.6% and 20.6% of the subjects, respectively. Fifteen transmission networks involving HCV (subtype 1a, 1b, 3a and 3b), HIV-1 (CRF33_01B) and HPgV (genotype 2) were identified and characterized. Genealogical estimates indicated that the predominant HCV, HIV-1 and HPgV genotypes were introduced into the IDUs population through multiple sub-epidemics that emerged as early as 1950s (HCV), 1980s (HIV-1) and 1990s (HPgV). By determining the difference in divergence times between viral lineages (ΔtMRCA), we also showed that the frequency of viral co-transmission is low among these IDUs. Despite increased access to therapy and other harm reduction interventions, the continuous emergence and coexistence of new transmission networks suggest persistent multiple viral transmissions among IDUs.

Hepatitis C virus (HCV) is a bloodborne virus from the *Hepacivirus* genus of the family *Flaviviridae*, commonly implicated to liver cirrhosis and hepatocellular carcinoma in humans^1^. The positive-sense RNA virus continues to cause severe morbidity and mortality among people who are infected through intravenous drug use, contaminated blood products and unsafe medical procedures. To date, there are six confirmed genotypes denoted as genotype 1 through 6 and a provisionally assigned genotype 7^2^,^3^, with each genotype diverging further into different subtypes with distinct genetic variability. Due to the shared transmission route, co-infection of human immunodeficiency virus type 1 (HIV-1) and human pegivirus (HPgV, formerly known as GB virus C or hepatitis G virus) among HCV-infected individuals are common, especially among people who inject drugs^4^.

HIV-1 is known for its high genetic diversity and persistent nature of infection^5^. Recent successes and greater accessibility to combination antiretroviral therapy has led to substantial reduction in mortality from AIDS-related opportunistic infections and malignancy globally^6^. However, treatment can be challenging in the context of HCV co-infection, largely because HIV-1-infected individuals are less responsive to HCV treatment with peg-interferon and ribavirin, and are at a higher risk of hepatotoxicity and drug interactions^7^. Consequently, this has led the emergence of liver pathology as a cause of morbidity and mortality in HIV-1 patients co-infected with HCV^8^.

HPgV is a member of the *Pegivirus* genus in the *Flaviviridae* family that is known to infect humans, but is apparently non-pathogenic or of very low pathogenic potential^9^. Several studies suggested that HPgV infections among HIV-1-infected individuals may yield favourable clinical outcomes such as higher CD4^+^ cell counts, lower HIV-1 viral loads, slower disease progression, and longer survival term^10^. Conversely, in HCV-infected individuals, studies have indicated that HPgV infection is likely to be associated with slower HCV clearance, leading to a higher likelihood of persistent infection^11^.

Although previous investigations have highlighted the clinical significance and epidemiological impact of viral co-infections^7^,^8^,^11^, co-analysis on the evolutionary dynamics and transmission network profiles of HCV, HIV-1 and HPgV within a single cohort remains limited, especially among individuals with multiple infections. Phylogenetic analysis using viral genetic sequence has been proven useful in assessing and defining transmission networks within a population^12^. Studies on HIV-1 have highlighted the potential role of transmission networks in fuelling the global epidemic^13^,^14^. However, the frequency and profiles of HCV and HPgV transmission networks remains largely uninvestigated, particularly in the context of co-infections. As a result, data on shared transmission networks that may possibly indicate co-transmission of HCV, HIV-1 and/or HPgV are lacking. To this aim, we attempted to identify the transmission networks and place a genetic timescale on the population history of HCV, HIV-1 and HPgV circulating among a cohort of injecting drug users (IDUs) in Malaysia. Using network information and divergence time estimates, we deduce the possibility of viral co-transmission among individuals with multiple infections.

## Results

### HCV, HIV-1 and HPgV co-infections and subtypes distribution among people who inject drugs in Kuala Lumpur, Malaysia

**Figure 1:**
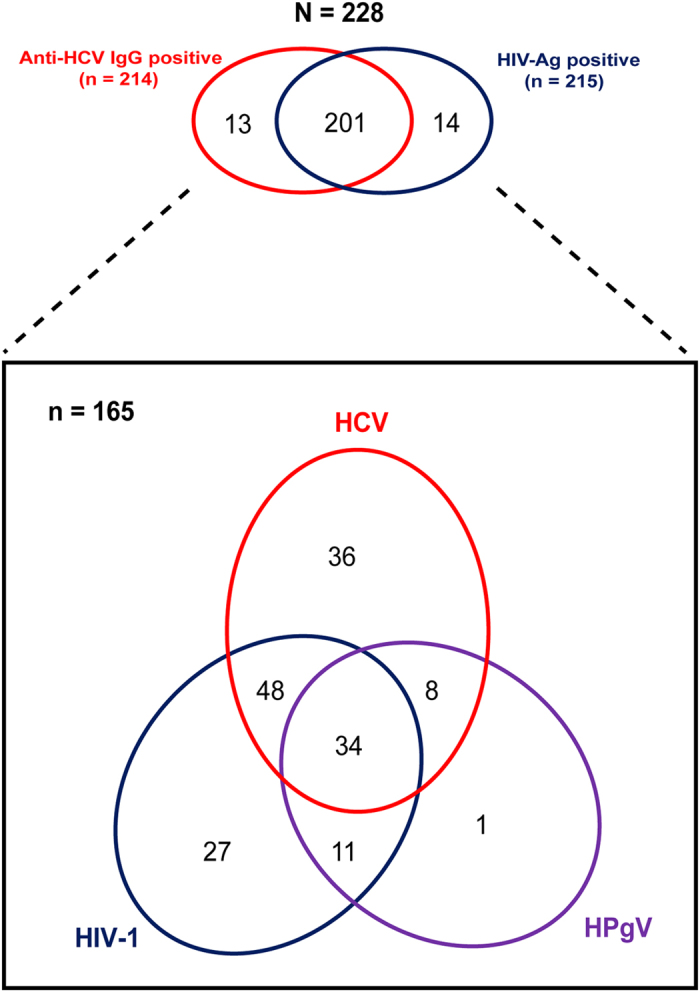
Schematic representation of mono- and co-infection cases among people who inject drugs in Kuala Lumpur. HCV and HIV-1 seropositivity was estimated at 94% each. Nested PCR of the 5′-UTR and NS5B gene of both HCV and HPgV as well as the *gag-pol* gene of HIV-1 for all 228 individuals were performed, in which 165 subjects were positive for at least one target region. Red ellipse represents HCV infection, whereas blue and purple ellipses represent HIV-1 and HPgV infection, respectively. The overlap regions represent the viral co-infection cases.

A total of 228 IDUs, who were either positive for HCV (93.9%; 214/228) or HIV-1 (94.3%; 215/228) were recruited between September 2009 and November 2010 (Fig. 1). Data on the time for first positive HCV and HIV-1 serological tests for these subjects were not available. HCV/HIV-1 co-infection was detected in 88.2% of the individuals (201/228). Nested PCR of the 5′-UTR and NS5B gene of HCV and the *gag-pol* gene of HIV-1 were performed for seropositive samples for HCV and HIV-1, respectively. HPgV seroprevalence was not determined due to the lack of a commercially available serology assay. Therefore, nested PCR of the 5′-UTR and NS5B of HPgV for all 228 individuals were conducted. A total of 165 subjects were positive for at least one target region. Based on the availability of the sequence data, mono-infection was detected in 38.8% (64/165) of the subjects (HCV = 36, HIV-1 = 27, HPgV = 1). Cases of dual-infection were detected in 40.6% (67/165) individuals (HCV/HIV = 48, HCV/HPgV = 8, HIV/HPgV = 11). HCV/HIV/HPgV triple-infection was detected in 20.6% (34/165) of study subjects (Fig. 1).

From 126 PCR-positive HCV-infected individuals, phylogenetic analysis of the 5′-UTR and NS5B gene showed that subtype 3a was the predominant strain at 46.0% (58/126), followed by subtype 1a (31.0%, 39/126), 3b (11.1%, 14/126), 1b (10.3%, 13/126) and 6n (1.6%, 2/126) (Supplementary Figure S1). Subtype assignment in both the 5′-UTR and NS5B was concordant. Neighbour-joining inference of the *gag-pol* (or the *protease*) regions of HIV-1 in 120 individuals showed the predominance of CRF33_01B at 76.7% (92/120). This is followed by HIV-1 subtype B′ (a Thai variant of subtype B) (13.3%, 16/120), CRF01_AE (5.0%, 6/120) and other minor lineages such as CRF53_01B, CRF58_01B and unique recombinants at 5.0% (6/120) (Supplementary Figure S2). In the absence of HPgV serological data, phylogenetic estimates of the 5′-UTR and NS5B gene in 54 subjects revealed that genotypes 2, 3 and 4 were circulating at 79.6% (43/54), 16.7% (9/54) and 3.7% (2/54), respectively (Supplementary Figure S3). Discordance in genotype assignment between the 5′-UTR and NS5B trees was not observed. Chi-square analysis indicated no significant correlations were observed in the distribution of HCV, HIV-1 and HPgV genotypes among the multiply infected subjects (*P* > 0.05) (Table 1).

### Transmission networks and evolutionary histories

**Figure 2:**
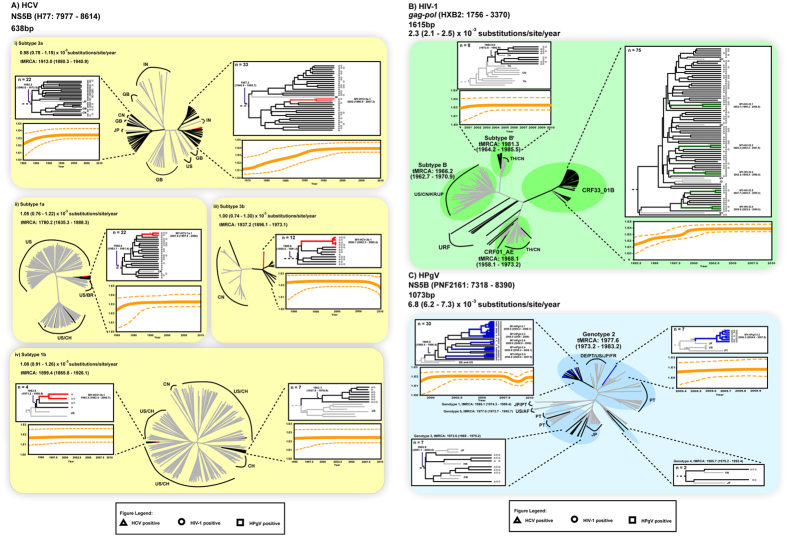
Phylodynamic profiles of HCV, HIV-1 and HPgV among people who inject drugs in Kuala Lumpur, Malaysia. (**A)** Bayesian maximum clade credibility (MCC) tree of 638 bp HCV NS5B gene (H77: 7977–8614), **(B)** 1615 bp HIV-1 *gag-pol* gene (HXB2: 1756–3370) and **(C)** 1073 bp HPgV NS5B (PNF2161: 7318–8390) sequence data collected between September 2009 and November 2010 are shown. The transmission networks observed within HCV, HIV-1 and HPgV lineages are colour-coded accordingly. Black branches are unique transmission lineages that do not cluster, while gray branches are reference sequences. The Bayesian coalescent-based relaxed molecular clock model was performed in BEAST 1.7, with uncorrelated lognormal model nested in general time-reversible (GTR) nucleotide substitution model and a proportion of invariant sites for all three viruses. The Markov chain Monte Carlo (MCMC) analysis was computed four times at 50 million states sampled every 10,000 states and output was assessed for convergence by means of effective sampling size after a 10% burn-in. Transmission networks are defined based on strong statistical supports generated at the internal nodes of the maximum likelihood and MCC tree reconstructions (bootstrap values of more than 90% and posterior probability of 1.0, respectively) and genetic distance of ≤0.025. A total of 4, 5 and 6 monophyletic transmission networks were observed within HCV, HIV-1 ad HPgV, respectively. The symbols at the tip of each branch represent the status of infection. Country abbreviations: US: United States of America, CN: China, MY: Malaysia, CH: Switzerland, JP: Japan, PT: Portugal, BR: Brazil, GB: United Kingdom, IN: India, TH: Thailand, DE: Germany, FR: France, IN: Indonesia, HK: Hong Kong and AF: Africa.

To determine the frequency and size of HCV, HIV-1 and HPgV transmission networks, 107 HCV NS5B sequences, 94 HIV-1 *gag-pol* sequences and 46 HPgV NS5B sequences were further analyzed in PAUP and BEAST. Based on the criteria defined in this study (see Materials and Methods), transmission networks were identified among the HCV (10.6%, 10/94), HIV-1 (9.3%, 10/107) and HPgV (60.9%, 28/46) lineages. Phylogenetic reconstructions uncovered four HCV transmission networks (involving 2–4 subjects) in the Bayesian MCC trees, one each in subtypes 3a (MY-HCV-3a.1), 1a (MY-HCV-1a.1), 3b (MY-HCV-3b.1) and 1b (MY-HCV-1b.1) circulating in the population (Fig. 2A). Similar analysis revealed five HIV-1 transmission networks (involving 2 subjects each) within the CRF33_01B lineage (denoted MY-HIV-33.1 through MY-HIV-33.5) (Fig. 2B) and six HPgV transmission networks (involving 2–10 subjects) within the genotype 2 lineage (denoted as MY-HPgV-2.1 to MY-HPgV-2.6) (Fig. 2C). Maximum likelihood phylogenies reconstructed by PAUP also revealed the similar transmission networks.

**Figure 3:**
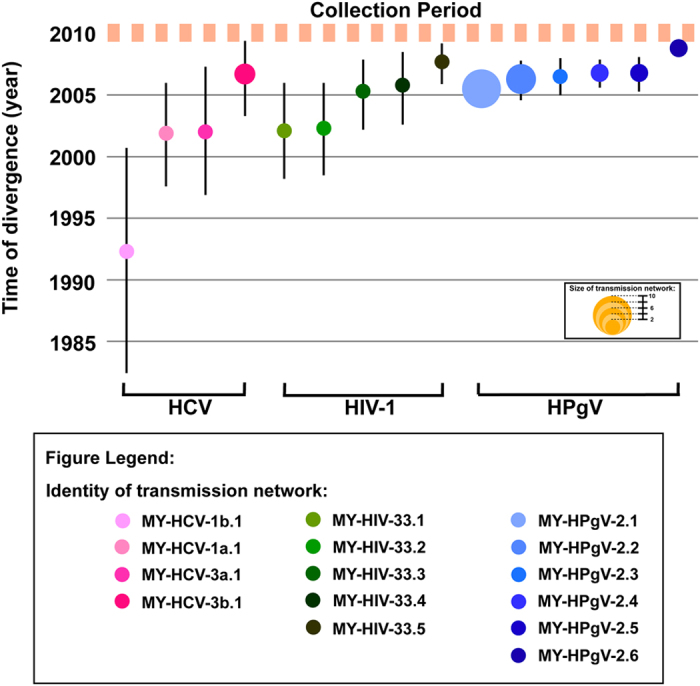
Divergence times of HCV, HIV-1 and HPgV transmission networks among people who inject drugs in Kuala Lumpur, Malaysia. The divergence time (in calendar years) of the most recent common ancestor and the 95% highest posterior distribution for the HCV transmission networks (red circles) observed among people who inject drugs in Kuala Lumpur, Malaysia were estimated based on NS5B gene (H77: 7977–8614, 638 bp) using Bayesian coalescent relaxed clock-based analysis in BEAST software and a general time-reversible nucleotide substitution model with a gamma distribution and a proportion of invariant sites. Under the similar phylogenetic settings, the origin of the HIV-1 (green circles) and HPgV (blue circles) transmission networks were estimated based on *gag-pol* gene (HXB2: 1756–3370, 1615 bp) and NS5B gene (PNF2161: 7318–8390, 1073 bp), respectively. The orange dotted line represents the period of sample collection, while the inset indicates the identity and size of each transmission network.

Bayesian analyses of the time-stamped NS5B gene of HCV (n = 964) and the HIV-1 *gag-pol* gene (n = 226) sequences under the relaxed clock model (uncorrelated lognormal) with constant population size revealed a mean evolutionary rates of 0.98–1.08 (0.74–1.30) × 10^−3^ substitutions/site/year (across subtypes 3a, 1a, 3b and 1b) and 2.3 (2.1–2.5) × 10^−3^ substitutions/site/year, comparable with estimates published previously^15^,^16^. Based on these evolutionary *a priori*, the divergence time for HCV subtypes were dated around the mid 1700s to mid 1900s, in line with estimates reported elsewhere^17^,^18^. There were two distinct 3a lineages circulating in Malaysia since the 1950s that possibly represent the earliest/oldest lineages in the country. Likewise, the major subtype 1a lineage in the country was dated back to the 1960s, and was closely related to the United States/Brazil (US/BR) strains. Sporadic introduction (or occasional spread) of subtype 1a lineages related to the United States/Switzerland (US/CH) strains was also observed. Subtype 1b lineage closely related to the CH strains was dated around the 1960s too, with a more recent but genetically distinct lineage related to the US/CH isolates introduced into the region in the 1980s. Around the same period, subtype 3b lineage was established in the country. In addition, the time of the most recent common ancestor (tMRCA) of each HCV transmission networks was estimated to be around the early 1990s to mid 2000s, with the oldest network involving subtype 1b emerging as early as in the 1980s (Fig. 2A). Of note, more recent networks that were detected thereafter involved the major circulating HCV subtypes 3a, 1a and 3b (Fig. 3). Similarly, the origin of HIV-1 subtypes B, B′ and CRF01_AE dated back to between the late 1960s and mid 1980s (Fig. 2B), similar to the dates estimated in other studies^19^,^20^,^21^,^22^,^23^. The tMRCA of each HIV-1 transmission network observed within CRF33_01B was dated between early 2000s to late 2000s (Fig. 3). For HPgV, the evolutionary rate of the NS5B gene was newly estimated at 6.8 (6.2–7.3) × 10^−3^ substitutions/site/year, an order of magnitude difference from the rate estimated elsewhere^24^. The divergence time of HPgV were dated more recently than that of HCV and HIV-1, with HPgV genotypes 2, 3 and 5 were first dated around the 1970s, followed by genotypes 1 and 4 (1980s) (Fig. 2C). Two distinct HPgV genotype 2 lineages were observed, one of which emerged around mid 1990s that might represent the first genotype 2 lineage circulating in the country. Following HPgV introduction, multiple genotype 2 transmission networks continued to spread among the IDUs population. All HPgV transmission networks observed within genotype 2 were dated around the mid to late 2000s (Fig. 3).

### Difference in divergence times (ΔtMRCA) between viral lineages within individual

**Figure 4:**
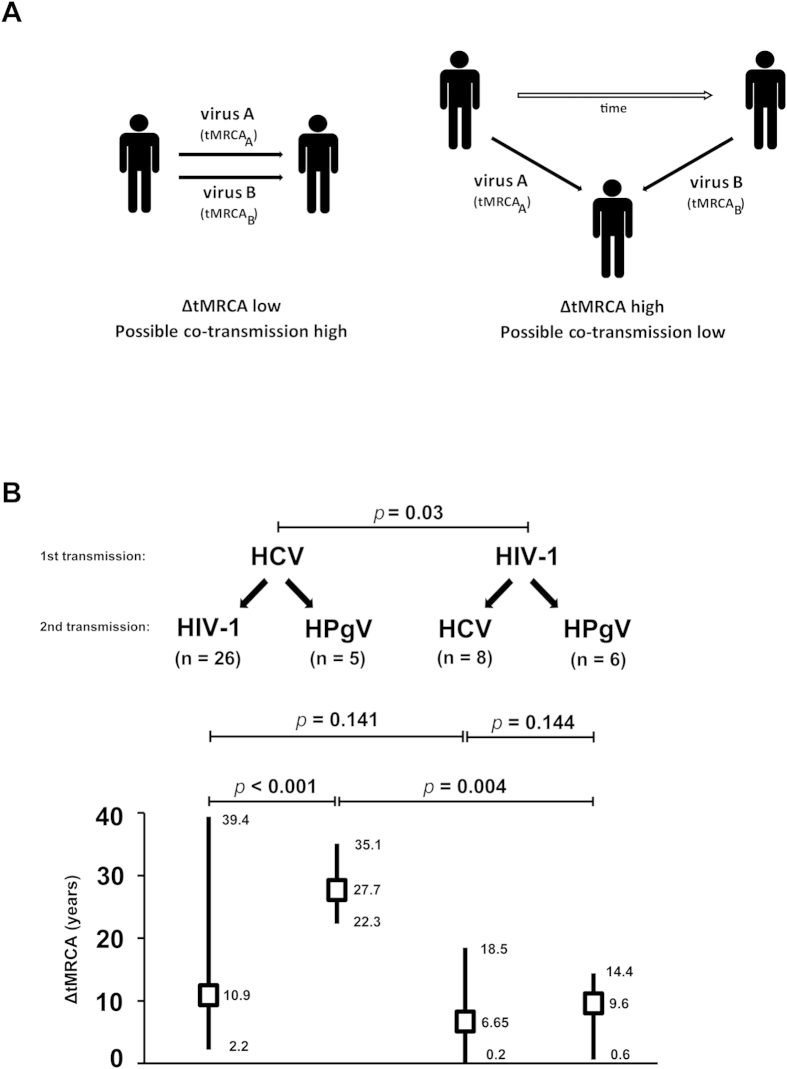
The difference in divergence time (ΔtMRCA) between HCV, HIV-1 and HPgV lineages among individuals with multiple viral infections in Kuala Lumpur, Malaysia. (**A**) Two possible scenario of viral transmission is illustrated. When two viruses are co-transmitted to an individual at a particular point of time, the divergence times of the co-transmitted lineages will be similar, thus the low ΔtMRCA. However, when two viruses are transmitted to an individual at two distinct time points, the estimated ΔtMRCA will be high. (**B**) The impact of HCV and HIV-1 infection on the duration in acquiring subsequent viral infections is shown. The estimated ΔtMRCAs were analyzed using the non-parametric Mann-Whitney U test statistical analysis, of which *P* < 0.05 is considered significant.

We attempted to investigate the frequency of viral co-transmission (or viral co-acquisition) among the co-infected subjects by means of inferring the time of origin or divergence time of viral lineages using the Bayesian coalescent method described above. In the absence of the first seropositive dates in our cohort, we estimated the duration between two transmission events by measuring the difference of the mean divergence times between two viral lineages (ΔtMRCA) within a dually infected subject. In this proof-of-concept approach, if two viruses were co-transmitted to a susceptible individual at a particular point of time, the divergence times of the co-transmitted lineages will be similar, thus the low ΔtMRCA (Fig. 4A). In contrast, if two viruses were transmitted to an individual at two distinct time points, the estimated ΔtMRCA will be high. In our data set, the dually infected subjects (n = 45, based on the availability of NS5B gene of both HCV and HPgV, and *gag*-*pol* gene of HIV-1) were grouped into two categories: a) HCV-infected subjects co-infected with either HIV-1 (n = 26) or HPgV (n = 5) and b) HIV-1-infected subjects co-infected with either HCV (n = 8) or HPgV (n = 6) (Fig. 4B). It was estimated that only one HIV-1/HCV co-infected patient (1/45, 2.2%) showed a ΔtMRCA of approximately 73 days, whereas in other subjects the calculated ΔtMRCAs in general exceeded 100 days, suggesting a low frequency of viral co-transmission among IDUs.

To test the impact of HCV and HIV-1 infection on the duration to acquire subsequent viral infections, the quantitative ΔtMRCA estimates were further analyzed by the non-parametric Mann-Whitney U test. The order of viral transmission was deduced based on the divergence times. It was estimated that subjects first infected with HIV-1 (n = 14) experienced a shorter time (lower ΔtMRCA) to acquire subsequent HCV or HPgV infections, as compared to the HCV-infected subjects (n = 31) who later acquired HIV-1 or HPgV infections (*P* = 0.03) (Fig. 4B). However, among the HIV-1-infected subjects who were then infected with either HCV or HPgV, no significant difference in ΔtMRCA was observed (*P* = 0.144). Among subjects first infected with HCV, the duration to acquire HPgV infection was significantly longer than that to acquire HIV-1 infection (*P* < 0.001) (Fig. 4B).

## Discussion

Transmission of bloodborne viruses such as HCV among people who inject drugs continues to cause major public-health problems worldwide. Due to the shared mode of transmission with HCV, co-infections of HIV-1 and HPgV among HCV-infected patients are common^25^,^26^. Although studies have indicated the clinical and epidemiological impact of co-infections involving these viruses, co-analysis on the evolutionary history and phylodynamic profiles of these viruses within a defined high-risk population remains limited. Several recent studies on HIV-1 among men who have sex with men have addressed the population dynamics of the virus, and pointed out the potential role of transmission networks in escalating HIV-1 transmission worldwide^13^,^14^,^27^,^28^. However, such features in HCV and HPgV infections have not been widely studied, particularly in the context of co-infections. In the present study, we analyzed the genetic history and the transmission networks of HCV, HIV-1 and HPgV within a cohort of individuals who inject drugs in Kuala Lumpur, Malaysia, and assessed the possible occurrence of viral co-transmission in the population using phylodynamic data.

Viral sequence data has been proven informative in defining transmission networks within a population, particularly those with limited epidemiological data such as sexual history and injecting drug use^15^,^29^. In addition, studies have also highlighted the usefulness and the importance of exploiting viral genetic information in assessing the epidemic linkages through viral phylodynamics studies^15^,^30^. A total of 107 HCV NS5B gene, 94 HIV-1 *gag-pol* gene and 46 HPgV NS5B gene sequence data were phylogenetically analyzed. Bayesian coalescent analysis estimated that HCV subtype 1a was first emerged around the 1700s, followed by 1b (1800s), 3a and 3b (1900s). It is estimated that the most prevalent HCV subtype 3a first introduced into the population who inject drugs in Kuala Lumpur, Malaysia through at least two distinct introductions dating back as early as in the 1950s. This period in the 20th century probably correlated with the practice of non-sterile medical procedures that could have facilitated viral transmission^31^. Bayesian skyline plot for HCV subtype 3a lineage indicated the initial growth phase started around mid-1970s and early 1980s, followed by an exponential growth and reached its peak from 1990s onwards. Shortly after the introduction of subtype 3a, other HCV subtypes were introduced into IDUs between the early 1960s and the early 1980s, and established a steady growth (Fig. 2A).

In addition, Bayesian coalescent analysis suggested that HIV-1 subtype B, B′ and CRF01_AE emerged between the late 1960s and early 1980s, corroborating with previous reports^19^,^20^,^21^,^22^,^23^. The Malaysian subtype B′ and CRF01_AE isolates were traced to the divergence times similar to that of the Thai reference strains, indicating their shared ancestry^32^,^33^,^34^. The data also suggest that HIV-1 infections among IDUs were primarily driven by HIV-1 CRF01_AE and subtype B′^35^. However, nearly 80% of study subjects were infected with HIV-1 CRF33_01B, with numerous transmission networks observed between the early to mid-2000s. Bayesian skyline analysis indicated that the primary growth phase was initiated nearly two decades ago and reached its peak around the early 2000s (Fig. 2B)^36^.

The divergence time of HPgV genotypes were estimated, suggesting more recent divergence times than those of HCV and HIV-1. HPgV genotypes exhibited distinct geographical distribution patterns, with genotypes 1 and 5 commonly found in Africa, genotype 2 in Europe and the United States, and genotypes 3 and 4 in Asia^37^. In the present study, the predominant HPgV genotype is genotype 2, suggesting an HPgV infiltration into the country through a link that was probably related to the West (Europe/United States). Bayesian skyline plot analysis suggested that the primary growth phase was initiated in the late 1990s and reached its plateau a decade later (Fig. 2C). To our knowledge, this is the first report documenting the evolutionary histories of global HPgV genotypes and their subsequent spread into Malaysia through intravenous drug use.

Studies on HIV-1 have highlighted the potential role of transmission networks in fuelling the global epidemic. In the present study, multiple transmission networks were observed (Fig. 3), emphasizing their role in shaping current disease burden among IDUs in Kuala Lumpur. Despite the increased access to antiretroviral therapy and other intervention measures more than a decade ago, new transmission networks continue to emerge thereafter. The present study suggests that the trend of continuous emergence of new transmission networks and coexistence of multiple IDUs subepidemics from various common ancestors are likely to cause continuous onward viral transmission.

We explored the unique approach of using the Bayesian coalescent estimates to deduce the frequency of viral co-transmission within a dually infected subject. In the absence of seroconversion data, the divergence time from a common ancestor of a virus lineage could represent the time of infection or transmission^38^. By measuring the difference in divergence times between two viral lineages (ΔtMRCA), we found that the occurrence of viral co-transmission among the IDUs population in Kuala Lumpur is low (Fig. 4B). However, the preliminary findings should be interpreted with care because the estimated tMRCA is simply an indication of the lineage divergence time, which somehow suggests the oldest date that a virus was transmitted. Actual viral transmission (and probably viral superinfection) could have also occurred at any time point between the estimated tMRCA and the date of collection. As such, the ΔtMRCA could have been mis-estimated, leading to false representation of co-transmission event. Primarily, our analysis also showed that HIV-1 infections may lead to a shorter time to acquire subsequent HCV or HPgV infections, compared to the HCV-infected subjects who later acquired HIV-1 or HPgV infections. It is possible that immune dysfunction such as impaired CD4 T-cell responses caused by HIV-1 infections may increase the risks to HCV infections^39^,^40^,^41^. However, such observation should be interpreted with caution due to the limited sample size studied, and potential case-reporting delay, as indicated by plateau of recent population size of the virus.

Several limitations have been identified in the present study. First, the short sampling period (2009–2010) might potentially underestimate the actual number of transmission networks. In addition, our analysis might not reveal the contemporary transmission patterns of these bloodborne viruses across international borders. This is because the existence of substantial sampling and publication biases could lead to overrepresentation of certain countries and under sampling from another^42^. In addition, the direction of transmission cannot be established from phylogenetic-based network because such transmission are not necessarily synonymous with actual transmission event^42^. Furthermore, the lack of commercially available assay for HPgV detection might have underestimated the actual prevalence of HPgV among IDUs.

To our knowledge, this is the first study that co-analyzed the genetic histories and transmission networks of HCV, HIV-1 and HPgV within a cohort of individuals with history of intravenous drug use, and assessed the characteristics of viral co-transmission in the population using phylodynamic data. Present study highlighted that the current disease burden observed among people who inject drugs is largely due to introduction of multiple HCV, HIV-1 and HPgV sub-epidemics lineages, originating few decades ago. Without effective preventive measures, continuous emergence of new transmission networks and co-existence of multiple sub-epidemics from various common ancestors are likely to fuel the HCV, HIV-1 and HPgV dissemination among people who inject drugs.

## Materials and Methods

### Ethics Statement

This study was approved by the University Malaya Medical Centre Medical Ethics Committee. Standard, multilingual consent forms validated by the Medical Ethics Committee were used. Written informed consent was obtained from all subjects prior to sample collection. All experiments were performed in accordance with relevant guidelines and regulations.

### Study Subjects and Specimens

In the present study, a total of 228 consenting IDUs, either HCV- and/or HIV-1-infected, were recruited in Kuala Lumpur, Malaysia between September 2009 and November 2010. Demographic information such as the age, gender and ethnicity were obtained, followed by collection of blood specimens for HCV and HIV-1 serological testing using Cobas 6000 (Roche Diagnostics, USA)^43^,^44^. HPgV seroprevalence was not determined due to the lack of commercially available serology assay.

### Viral RNA Extraction, Gene Amplification and Sequencing

Total viral RNA was extracted from 140 μl of plasma specimens using the NucliSENS easyMAG automated nucleic acid extraction system (bioMérieux, Marcy I’Etoile, France)^45^, as described in the manufacturer’s protocol. Subsequently, the viral RNA was reverse transcribed using the SuperScript III reverse transcriptase (Invitrogen, USA). Nested-PCR was performed using previously reported primers sets to amplify the *gag-pol* gene of HIV-1 (HXB2 position: 1756–3370, 1615 bp)^46^, 5′-untranslated region (UTR) and NS5B gene of both HCV (H77 position: 62–696, 635 bp and 7977–8614, 638 bp)^47^ and HPgV (PNF2161 position: 163–367, 204 bp and 7318–8390, 1073 bp)^48^,^49^. For HIV-1, the *protease* gene (HXB2 position: 1793–2581, 789 bp) will be amplified for subtype determination should the longer *gag-pol* gene was negative. The PCR products were sequenced by an ABI PRISM 3730XL DNA Analyzer (Applied Biosystems, USA). The nucleotide sequence data were then codon aligned along with the respective list of viral reference sequences retrieved from the Los Alamos National Laboratory HIV (www.hiv.lanl.gov) and HCV sequence database (www.hcv.lanl.gov) and GenBank (www.ncbi.nlm.nih.gov/genbank/).

### Phylogenetic Reconstructions, Phylodynamic Inference and Divergence Times of Transmission Networks

To determine the HCV, HIV-1 and HPgV genetic subtype and to identify the potential transmission networks, neighbour-joining trees were first reconstructed based on the 5′-UTR and NS5B gene of both HCV and HPgV, as well as the *gag-pol* gene of HIV-1 from the sequence data set using MEGA version 6.0 based on Kimura-2 parameter model^50^. Since the *gag-pol* gene and NS5B gene have been proven useful in assessing and defining transmission networks^31^,^51^, these genes were used for subsequent analysis. The reliability of phylogenies were further tested by a more robust maximum likelihood approach, heuristically inferred using subtree pruning and regrafting, as well as nearest neighbour interchange algorithms implemented in PAUP version 4.0^52^. The statistical significance of the branching orders was validated by bootstrap analysis of 1000 replicates. The most appropriate nucleotide substitution model was determined using FindModel, a web implementation of Modeltest. Bayesian maximum clade credibility (MCC) trees were constructed using BEAST 1.7^53^ to determine the posterior probability values for each network observed in HCV, HIV-1 and HPgV. Transmission network for HIV-1 is defined as a network consisting of at least 2 isolates from different individuals of the same geographical (i.e country) origin with a genetic distance of ≤ 0.025, which formed a monophyletic phylogenetic clade supported by bootstrap value of more than 90% and Bayesian posterior probability value of 1.0 at the internal tree node, as described previously^27^,^30^,^54^. Due to the lack of a definition for a phylogenetic transmission network for HCV and HPgV, similar classification used for HIV-1 was applied to these viruses.

The time of most recent common ancestor (tMRCA) of the respective transmission networks observed in each genomic region of HCV, HIV-1 and HPgV were estimated by the Bayesian coalescent-based relaxed molecular clock model, conducted in BEAST 1.7^53^. Previously reported HCV NS5B and HIV-1 *gag-pol* gene nucleotide substitution rates (1.0 × 10^−3^ and 2.0 × 10^−3^ substitutions/site/year, respectively)^15^,^16^ were used as *a priori*. Since the HPgV nucleotide substitution rates were previously estimated from a small data set sampled over a short study period^55^, we attempted to re-estimate the evolutionary rate based on all available HPgV NS5B sequences (n = 132) sampled between 1993 and 2011. For the coalescent priors, different parametric demographic models namely constant population size, exponential and logistic growth as well as nonparametric Bayesian skyline were tested. The best fit coalescent model was chosen by means of a Bayes factor (Supplementary Table S1). Accordingly, the constant population size and uncorrelated lognormal model^29^ nested in general time-reversible (GTR) nucleotide substitution model^56^ with a proportion of invariant sites and four rate categories of gamma-distribution model of among site rate heterogeneity^57^ was employed to estimate the HCV, HIV-1 and HPgV phylogenies, and to date the tMRCA of each transmission networks^31^,^51^. The Markov chain Monte Carlo (MCMC) analysis was computed four times at 50 million states sampled every 10,000 states and output was assessed for convergence by means of effective sampling size after a 10% burn-in using Tracer version 1.5 (http://tree.bio.ed.ac.uk/software/tracer).

### Sequences

HCV, HIV-1 and HPgV nucleotide sequences generated in the study have been deposited in GenBank under the accession numbers KR108391-KR108696, KR131788-KR131833 and KC477938-KC478065. Similar information can be found in Supplementary Table S2.

## Additional Information

**How to cite this article**: Ng, K.T. *et al.* Co-infections and transmission networks of HCV, HIV-1 and HPgV among people who inject drugs. *Sci. Rep.* **5**, 15198; doi: 10.1038/srep15198 (2015).

## Supplementary Material

Supplementary Information

## Figures and Tables

**Table 1:** The distributions of viral subtypes/genotypes in co-infection cases.

HIV-1	HCV					Total
	1a	1b	3a	3b	6n	
HCV/HIV-1 co-infection by subtypes						
Subtype B'	3	1	6	1	0	11
CRF01_AE	0	1	1	1	0	3
CRF33_01B	16	7	27	11	1	62
Others	2	1	2	1	0	6
Total	21	10	36	14	1	82
*P*-value > 0.05 (0.85)						

HCV/HPgV co-infection by subtypes						
**HPgV**						
Genotype 2	9	2	16	5	1	33
Genotype 3	3	0	3	2	0	8
Genotype 4	0	0	1	0	0	1
Total	12	2	20	7	1	42
*P*-value > 0.05 (0.83)						

HIV-1/HPgV co-infection by subtypes						
**HPgV**	**HIV-1**					
	**Subtype B'**	**CRF01_AE**	**CRF33_01B**	**Others**	**Total**	
Genotype 2	6	1	26	2	35	
Genotype 3	2	0	6	0	8	
Genotype 4	0	0	2	0	2	
Total	8	1	34	2	45	
*P*-value > 0.05 (0.71)						

## References

[b1] Hoofnagle J. H. Course and outcome of hepatitis C. Hepatology 36, S21–29 (2002). 12407573 10.1053/jhep.2002.36227

[b2] Simmonds P. *et al.* Classification of hepatitis C virus into six major genotypes and a series of subtypes by phylogenetic analysis of the NS-5 region. J Gen Virol 74, (Pt 11), 2391–2399 (1993). 8245854 10.1099/0022-1317-74-11-2391

[b3] Murphy D. G. *et al.* Use of sequence analysis of the NS5B region for routine genotyping of hepatitis C virus with reference to C/E1 and 5’ untranslated region sequences. J Clin Microbiol 45, 1102–1112 (2007). 17287328 10.1128/JCM.02366-06 PMC1865836

[b4] Marco A., Gallego C. & Cayla J. A. Incidence of hepatitis C infection among prisoners by routine laboratory values during a 20-year period. PLoS One 9, e90560 (2014). 24587394 10.1371/journal.pone.0090560 PMC3938777

[b5] Malim M. H. & Emerman M. HIV-1 sequence variation: drift, shift, and attenuation. Cell 104, 469–472 (2001). 11239404 10.1016/s0092-8674(01)00234-3

[b6] United Nations and AIDS. Joint United Nations Programme on HIV/AIDS. *UNAIDS report on the global AIDS epidemic*. http://www.unaids.org/en/media/unaids/contentassets/documents/epidemiology/2013/gr2013/UNAIDS_Global_Report_2013_en.pdf (2013) (Date of access: 13 August 2014).

[b7] Graham C. S. *et al.* Influence of human immunodeficiency virus infection on the course of hepatitis C virus infection: a meta-analysis. Clin Infect Dis 33, 562–569 (2001). 11462196 10.1086/321909

[b8] Ioannou G. N. *et al.* The prevalence of cirrhosis and hepatocellular carcinoma in patients with human immunodeficiency virus infection. Hepatology 57, 249–257 (2013). 22532055 10.1002/hep.25800

[b9] Heringlake S. *et al.* GB virus C/hepatitis G virus infection: a favorable prognostic factor in human immunodeficiency virus-infected patients? J Infect Dis 177, 1723–1726 (1998). 9607857 10.1086/517431

[b10] Alcalde R. *et al.* Prevalence and distribution of the GBV-C/HGV among HIV-1-infected patients under anti-retroviral therapy. Virus Res 151, 148–152 (2010). 20420864 10.1016/j.virusres.2010.04.008

[b11] Tenckhoff S. *et al.* Role of GB virus C in HIV-1-infected and hepatitis C virus-infected hemophiliac children and adolescents. J Acquir Immune Defic Syndr 61, 243–248 (2012). 23007118 10.1097/QAI.0b013e31826218e1. PMC3548052

[b12] Hue S., Clewley J. P., Cane P. A. & Pillay D. Investigation of HIV-1 transmission events by phylogenetic methods: requirement for scientific rigour. AIDS 19, 449–450 (2005). 10.1097/01.aids.0000161778.15568.a1 15750402

[b13] Ng K. T. *et al.* HIV-1 Transmission Networks Among Men Who Have Sex With Men in Asia. Clin Infect Dis 59, 910–911 (2014). 24944233 10.1093/cid/ciu480

[b14] Mayer K. H. Editorial commentary: The next tsunami? HIV spread in Asian men who have sex with men. Clin Infect Dis 58, 1760–1762 (2014). 10.1093/cid/ciu176 24647018

[b15] Hue S., Pillay D., Clewley J. P. & Pybus O. G. Genetic analysis reveals the complex structure of HIV-1 transmission within defined risk groups. Proc Natl Acad Sci USA 102, 4425–4429 (2005). 15767575 10.1073/pnas.0407534102 PMC555492

[b16] Gray R. R. *et al.* The mode and tempo of hepatitis C virus evolution within and among hosts. BMC Evol Biol 11, 131–141 (2011). 21595904 10.1186/1471-2148-11-131 PMC3112090

[b17] Li C., Lu L., Murphy D. G., Negro F. & Okamoto H. Origin of hepatitis C virus genotype 3 in Africa as estimated through an evolutionary analysis of the full-length genomes of nine subtypes, including the newly sequenced 3d and 3e. J Gen Virol 95, 1677–1688 (2014). 24795446 10.1099/vir.0.065128-0 PMC4103067

[b18] Lu L., Li C., Xu Y. & Murphy D. G. Full-length genomes of 16 hepatitis C virus genotype 1 isolates representing subtypes 1c, 1d, 1e, 1g, 1h, 1i, 1j and 1k, and two new subtypes 1m and 1n, and four unclassified variants reveal ancestral relationships among subtypes. J Gen Virol 95, 1479–1487 (2014). 24718832 10.1099/vir.0.064980-0 PMC4059268

[b19] Angelis K. *et al.* Global Dispersal Pattern of HIV Type 1 Subtype CRF01_AE: A Genetic Trace of Human Mobility Related to Heterosexual Sexual Activities Centralized in Southeast Asia. J Infect Dis 211, 1735–1744 (2014). 25512631 10.1093/infdis/jiu666

[b20] Li Z. *et al.* Tracing the origin and history of HIV-1 subtype B’ epidemic by near full-length genome analyses. AIDS 26, 877–884 (2012). 22269972 10.1097/QAD.0b013e328351430d

[b21] Li Y. *et al.* Explosive HIV-1 subtype B’ epidemics in Asia driven by geographic and risk group founder events. Virology 402, 223–227 (2010). 20435329 10.1016/j.virol.2010.03.048

[b22] Liao H. *et al.* Phylodynamic analysis of the dissemination of HIV-1 CRF01_AE in Vietnam. Virology 391, 51–56 (2009). 19540543 10.1016/j.virol.2009.05.023

[b23] Tee K. K. *et al.* Estimating the date of origin of an HIV-1 circulating recombinant form. Virology 387, 229–234 (2009). 19272628 10.1016/j.virol.2009.02.020

[b24] Romano C. M., Zanotto P. M. & Holmes E. C. Bayesian coalescent analysis reveals a high rate of molecular evolution in GB virus C. J Mol Evol 66, 292–297 (2008). 18320258 10.1007/s00239-008-9087-3 PMC3324782

[b25] Kouyos R. D. *et al.* Clustering of HCV coinfections on HIV phylogeny indicates domestic and sexual transmission of HCV. Int J Epidemiol 43, 887–896 (2014). 24453237 10.1093/ije/dyt276

[b26] Trinks J. *et al.* Human pegivirus molecular epidemiology in Argentina: Potential contribution of Latin American migration to genotype 3 circulation. J Med Virol 86, 2076–2083 (2014). 24615742 10.1002/jmv.23918

[b27] Zehender G. *et al.* Population dynamics of HIV-1 subtype B in a cohort of men-having-sex-with-men in Rome, Italy. J Acquir Immune Defic Syndr 55, 156–160 (2010). 20703157 10.1097/QAI.0b013e3181eb3002

[b28] Brenner B. G. *et al.* Transmission clustering drives the onward spread of the HIV epidemic among men who have sex with men in Quebec. J Infect Dis 204, 1115–1119 (2011). 21881127 10.1093/infdis/jir468 PMC3164430

[b29] Drummond A. J., Ho S. Y., Phillips M. J. & Rambaut A. Relaxed phylogenetics and dating with confidence. PLoS Biol 4, e88 (2006). 16683862 10.1371/journal.pbio.0040088 PMC1395354

[b30] Aldous J. L. *et al.* Characterizing HIV transmission networks across the United States. Clin Infect Dis 55, 1135–1143 (2012). 22784872 10.1093/cid/cis612 PMC3529609

[b31] Pybus O. G. *et al.* Genetic history of hepatitis C virus in East Asia. J Virol 83, 1071–1082 (2009). 18971279 10.1128/JVI.01501-08 PMC2612398

[b32] Deng X., Liu H., Shao Y., Rayner S. & Yang R. The epidemic origin and molecular properties of B’: a founder strain of the HIV-1 transmission in Asia. AIDS 22, 1851–1858 (2008). 18753865 10.1097/QAD.0b013e32830f4c62

[b33] Hemelaar J. The origin and diversity of the HIV-1 pandemic. Trends Mol Med 18, 182–192 (2012). 22240486 10.1016/j.molmed.2011.12.001

[b34] Korber B. *et al.* Timing the ancestor of the HIV-1 pandemic strains. Science 288, 1789–1796 (2000). 10846155 10.1126/science.288.5472.1789

[b35] Tee K. K., Saw T. L., Pon C. K., Kamarulzaman A. & Ng K. P. The evolving molecular epidemiology of HIV type 1 among injecting drug users (IDUs) in Malaysia. AIDS Res Hum Retroviruses 21, 1046–1050 (2005). 16379608 10.1089/aid.2005.21.1046

[b36] Chow W. Z. *et al.* Molecular diversity of HIV-1 among people who inject drugs in Kuala Lumpur, Malaysia: massive expansion of circulating recombinant form (CRF) 33_01B and emergence of multiple unique recombinant clusters. PLoS One 8, e62560 (2013). 23667490 10.1371/journal.pone.0062560 PMC3646884

[b37] Feng Y. *et al.* A novel genotype of GB virus C: its identification and predominance among injecting drug users in Yunnan, China. PLoS One 6, e21151 (2011). 21998624 10.1371/journal.pone.0021151 PMC3188531

[b38] de Oliveira T. *et al.* Molecular epidemiology: HIV-1 and HCV sequences from Libyan outbreak. Nature 444, 836–837 (2006). 17171825 10.1038/444836a

[b39] Beld M. *et al.* Evidence that both HIV and HIV-induced immunodeficiency enhance HCV replication among HCV seroconverters. Virology 244, 504–512 (1998). 9601518 10.1006/viro.1998.9130

[b40] Schnuriger A. *et al.* Acute hepatitis C in HIV-infected patients: rare spontaneous clearance correlates with weak memory CD4 T-cell responses to hepatitis C virus. AIDS 23, 2079–2089 (2009). 19710595 10.1097/QAD.0b013e328330ed24

[b41] Bailey J. R. *et al.* CD4+ T-Cell-Dependent Reduction in Hepatitis C Virus-Specific Neutralizing Antibody Responses After Coinfection With Human Immunodeficiency Virus. J Infect Dis 212, 914–923 (2015). 25754978 10.1093/infdis/jiv139 PMC4548462

[b42] Wertheim J. O. *et al.* The Global Transmission Network of HIV-1. J Infect Dis 209, 304–313 (2013). 24151309 10.1093/infdis/jit524 PMC3873788

[b43] Kesli R., Ozdemir M., Kurtoglu M. G., Baykan M. & Baysal B. Evaluation and comparison of three different anti-hepatitis C virus antibody tests based on chemiluminescence and enzyme-linked immunosorbent assay methods used in the diagnosis of hepatitis C infections in Turkey. J Int Med Res 37, 1420–1429 (2009). 19930846 10.1177/147323000903700516

[b44] Tao C. M. *et al.* Validation of the Elecsys(R) HIV combi PT assay for screening and reliable early detection of HIV-1 infection in Asia. J Clin Virol 58, 221–226 (2013). 23809476 10.1016/j.jcv.2013.05.012

[b45] Boom R. *et al.* Rapid and simple method for purification of nucleic acids. J Clin Microbiol 28, 495–503 (1990). 1691208 10.1128/jcm.28.3.495-503.1990 PMC269651

[b46] Tee K. K. *et al.* Identification of a novel circulating recombinant form (CRF33_01B) disseminating widely among various risk populations in Kuala Lumpur, Malaysia. J Acquir Immune Defic Syndr 43, 523–529 (2006). 17031320 10.1097/01.qai.0000242451.74779.a7

[b47] Lee Y. M. *et al.* Molecular epidemiology of HCV genotypes among injection drug users in Taiwan: Full-length sequences of two new subtype 6w strains and a recombinant form_2b6w. J Med Virol 82, 57–68 (2010). 19950240 10.1002/jmv.21658

[b48] Jarvis L. M. *et al.* Infection with hepatitis G virus among recipients of plasma products. Lancet 348, 1352–1355 (1996). 8918279 10.1016/s0140-6736(96)04041-x

[b49] Parreira R., Branco C., Piedade J. & Esteves A. GB virus C (GBV-C) evolutionary patterns revealed by analyses of reference genomes, E2 and NS5B sequences amplified from viral strains circulating in the Lisbon area (Portugal). Infect Genet Evol 12, 86–93 (2012). 22051938 10.1016/j.meegid.2011.10.011

[b50] Tamura K., Stecher G., Peterson D., Filipski A. & Kumar S. MEGA6: Molecular Evolutionary Genetics Analysis version 6.0. Mol Biol Evol 30, 2725–2729 (2013). 24132122 10.1093/molbev/mst197 PMC3840312

[b51] Hue S., Clewley J. P., Cane P. A. & Pillay D. HIV-1 pol gene variation is sufficient for reconstruction of transmissions in the era of antiretroviral therapy. AIDS 18, 719–728 (2004). 15075506 10.1097/00002030-200403260-00002

[b52] Swofford D. L. PAUP*: phylogenetic analysis using parsimony (*and other methods), version 4 beta. Sinauer Associates, Sunderland, MA (2003).

[b53] Drummond A. J. & Rambaut A. BEAST: Bayesian evolutionary analysis by sampling trees. BMC Evol Biol 7, 214–222 (2007). 17996036 10.1186/1471-2148-7-214 PMC2247476

[b54] Audelin A. M. *et al.* Phylogenetics of the Danish HIV epidemic: the role of very late presenters in sustaining the epidemic. J Acquir Immune Defic Syndr 62, 102–108 (2013). 23075917 10.1097/QAI.0b013e318276becc

[b55] Suzuki Y. *et al.* Slow evolutionary rate of GB virus C/hepatitis G virus. J Mol Evol 48, 383–389 (1999). 10079276 10.1007/pl00006482

[b56] Rodriguez F., Oliver J. L., Marin A. & Medina J. R. The general stochastic model of nucleotide substitution. J Theor Biol 142, 485–501 (1990). 2338834 10.1016/s0022-5193(05)80104-3

[b57] Yang Z. Maximum likelihood phylogenetic estimation from DNA sequences with variable rates over sites: approximate methods. J Mol Evol 39, 306–314 (1994). 7932792 10.1007/BF00160154

