# Supplementary material for: Co-infections and transmission networks of HCV, HIV-1 and HPgV among people who inject drugs
Source: Sci Rep. 2015 Oct 13;5:15198. doi: 10.1038/srep15198 (PMC4602306; doi:10.1038/srep15198)
Supplement: Supplementary Information [file srep15198-s1.pdf]

Supplementary Figure S1

Co-infections and transmission networks of HCV, HIV-1 and HPgV among people who inject drugs

Kim Tien Ng, Yutaka Takebe, Jack Bee Chook, Wei Zhen Chow, Kok Gan Chan, Haider Abdulrazzaq Abed Al-Darraji, Adeeba Kamarulzaman, and Kok Keng Tee

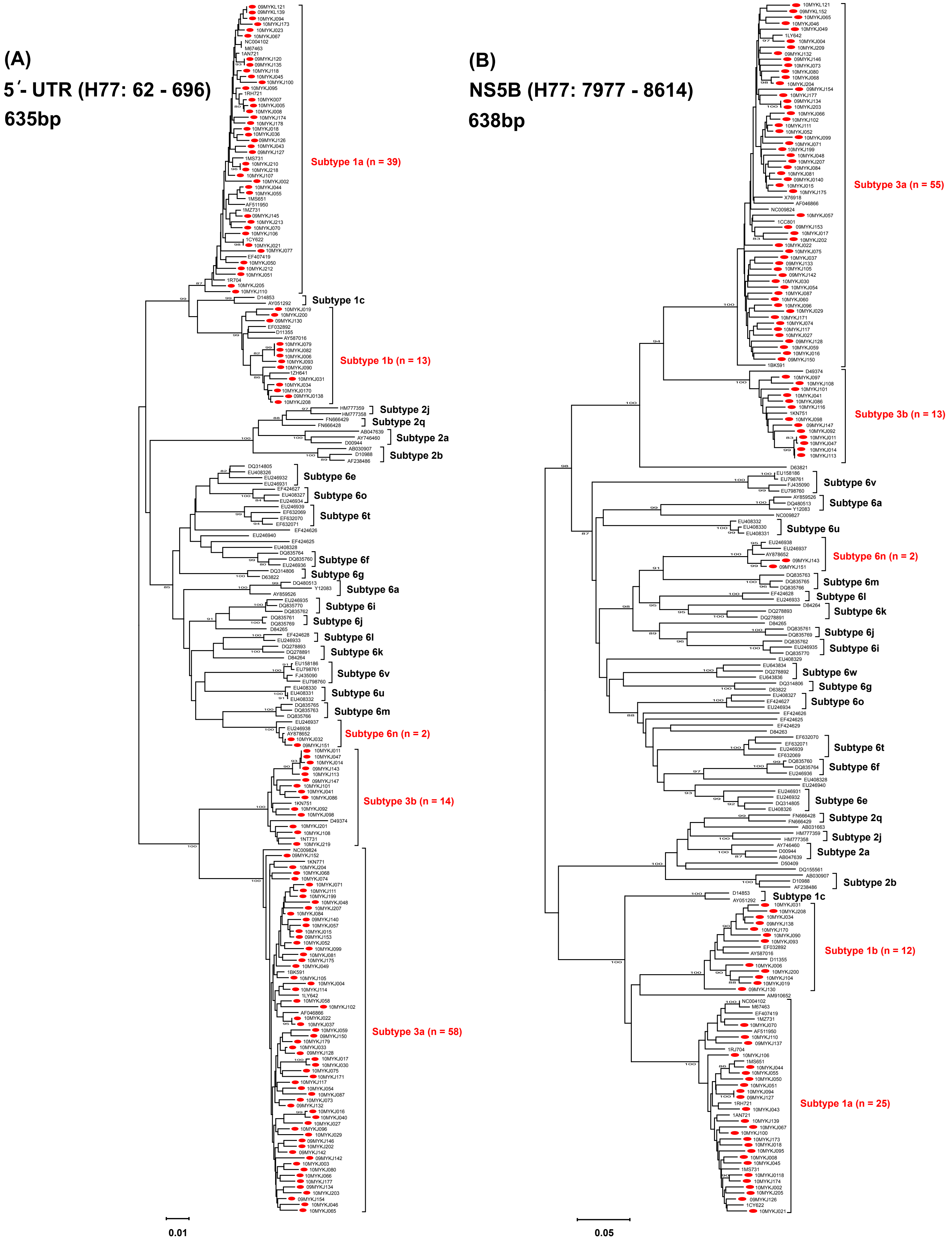

Supplementary Figure 1. HCV genotypes among people who inject drugs (red circles) in Kuala Lumpur, Malaysia. Neighbour-joining reconstruction based on (A) 5′-UTR region (H77: 62-696) and (B) NS5B gene (H77: 7977-8614) of HCV are shown. The statistical significance of the branching orders was validated by bootstrap analysis of 1000 replicates using MEGA version 6.0 based on Kimura-2 parameter model.

Supplementary Figure S2

Co-infections and transmission networks of HCV, HIV-1 and HPgV among people who inject drugs

Kim Tien Ng, Yutaka Takebe, Jack Bee Chook, Wei Zhen Chow, Kok Gan Chan, Haider Abdulrazzaq Abed Al-Darraji, Adeeba Kamarulzaman, and Kok Keng Tee

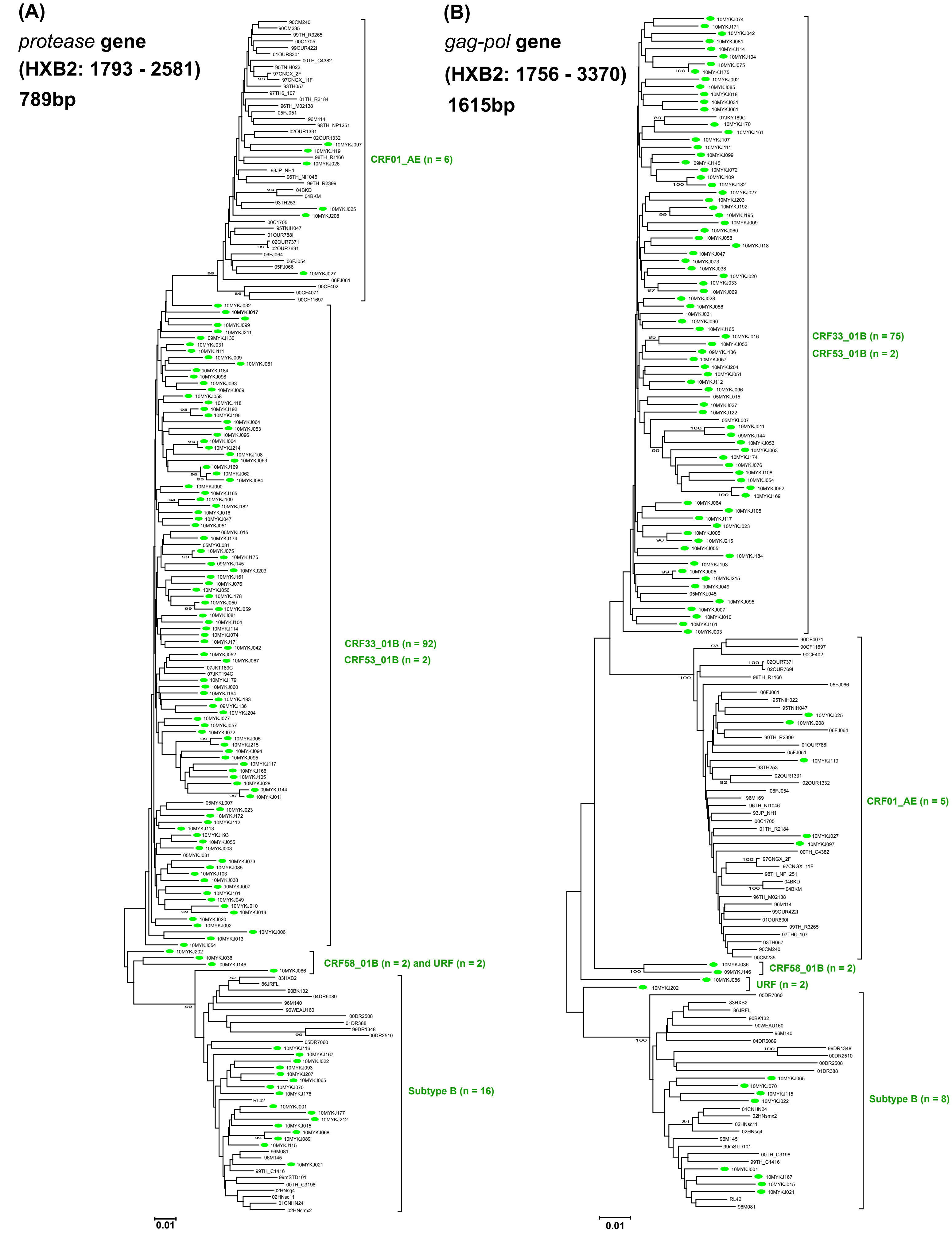

Supplementary Figure 2. HIV-1 genotypes among people who inject drugs (green circles) in Kuala Lumpur, Malaysia. Neighbour-joining reconstruction based on (A) *protease* gene (HXB2: 1793-2581) and (B) *gag-pol* gene (HXB2: 1756-3370) of HIV-1 are shown. The statistical significance of the branching orders was validated by bootstrap analysis of 1000 replicates using MEGA version 6.0 based on Kimura-2 parameter model.

Supplementary Figure S3

Co-infections and transmission networks of HCV, HIV-1 and HPgV among people who inject drugs

Kim Tien Ng, Yutaka Takebe, Jack Bee Chook, Wei Zhen Chow, Kok Gan Chan, Haider Abdulrazzaq Abed Al-Darraji, Adeeba Kamarulzaman, and Kok Keng Tee

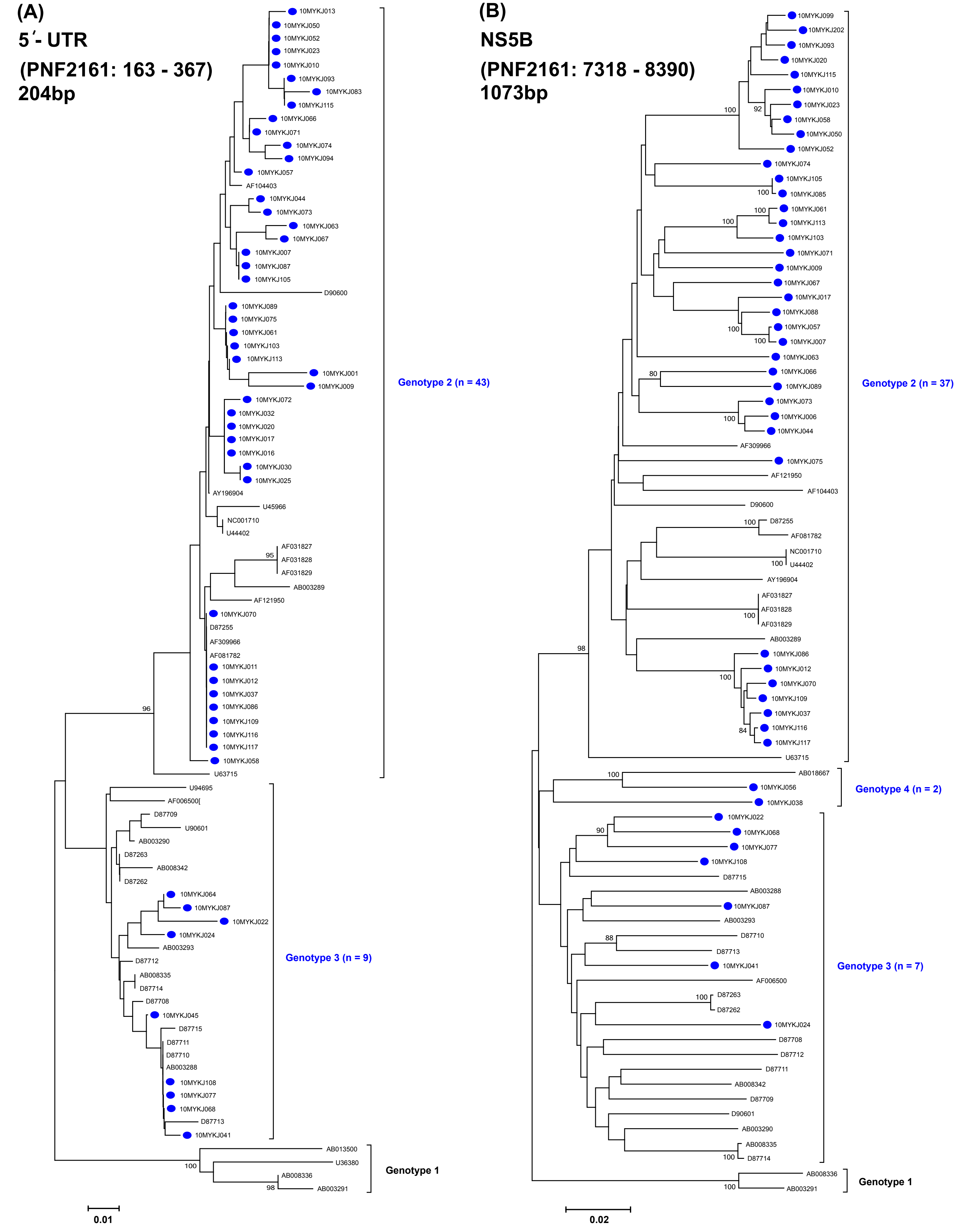

Supplementary Figure 3. HPgV genotypes among people who inject drugs (blue circles) in Kuala Lumpur, Malaysia. Neighbour-joining reconstruction based on (A) 5′-UTR region (PNF2161: 163-367) and (B) NS5B gene (PNF2161: 7318-8390) of HPgV are shown. The statistical significance of the branching orders was validated by bootstrap analysis of 1000 replicates using MEGA version 6.0 based on Kimura-2 parameter model.

Supplementary Table S1

Co-infections and transmission networks of HCV, HIV-1 and HPgV among people who inject drugs

Kim Tien Ng, Yutaka Takebe, Jack Bee Chook, Wei Zhen Chow, Kok Gan Chan, Haider Abdulrazzaq Abed Al-Darraji, Adeeba Kamarulzaman, and Kok Keng Tee

Supplementary Table S1: Comparison of different parametric and nonparametric demographic models by means of natural logarithm of Bayes factor (lnBF)

HCV

Model	Constant	Exponential	Logistic	Skyline
**Constant**	-	379.952	116.61	61.479
**Exponential**	-379.952	-	-207.184	-318.473
**Logistic**	-116.61	207.184	-	230.874
**Skyline**	-61.479	318.473	-230.874	-

HIV-1

Model	Constant	Exponential	Logistic	Skyline
**Constant**	-	56.257	71.001	145.19
**Exponential**	-56.257	-	43.884	88.932
**Logistic**	-71.001	-43.884	-	46.299
**Skyline**	-145.19	-88.932	-46.299	-

HPgV

Model	Constant	Exponential	Logistic	Skyline
**Constant**	-	5.99	2.3299	3.255
**Exponential**	-5.99	-	-2.9946	-2.735
**Logistic**	-2.3299	2.9946	-	2.8635
**Skyline**	-3.255	2.735	-2.8635	-

Bayes factors can be interpreted (Kass R & Raftery A, 1995. J Am Stat Assoc 90: 773-795) as follows:
2lnBF < 2 no evidence; 2 - 6 weak evidence; 6 - 10 strong evidence; > 10 very strong evidence

Kim Tien Ng, Yutaka Takebe, Jack Bee Chook, Wei Zhen Chow, Kok Gan Chan, Haider Abdulrazzaq Abed Al-Darraji, Adeeba Kamarulzaman, and Kok Keng Tee

Supplementary Table S2: Summary of patient ID, sample collection year and nucleotide accession number(s)

Virus	Patient ID	Collection Year	Accession number(s)
HCV	10MYKJ002	2010	KR108498, KR108391
HCV	10MYKJ008	2010	KR108501, KR108392
HCV	10MYKJ018	2010	KR108502, KR108393
HCV	10MYKJ021	2010	KR108503, KR108394
HCV	10MYKJ043	2010	KR108506, KR108395
HCV	10MYKJ044	2010	KR108507, KR108396
HCV	10MYKJ045	2010	KR108508, KR108397
HCV	10MYKJ050	2010	KR108509, KR108398
HCV	10MYKJ051	2010	KR108510, KR108399
HCV	10MYKJ055	2010	KR108511, KR108400
HCV	10MYKJ067	2010	KR108512, KR108401
HCV	10MYKJ070	2010	KR108513, KR108402
HCV	10MYKJ094	2010	KR108515, KR108403
HCV	10MYKJ095	2010	KR108516, KR108404
HCV	10MYKJ100	2010	KR108517, KR108405
HCV	10MYKJ106	2010	KR108518, KR108406
HCV	10MYKJ110	2010	KR108520, KR108407
HCV	10MYKJ118	2010	KR108521, KR108408
HCV	09MYKJ126	2009	KR108524, KR108409
HCV	09MYKJ127	2009	KR108525, KR108410
HCV	09MYKJ139	2009	KR108527, KR108412
HCV	10MYKJ173	2010	KR108529, KR108413
HCV	10MYKJ174	2010	KR108530, KR108414
HCV	10MYKJ205	2010	KR108532, KR108415
HCV	10MYKJ006	2010	KR108537, KR108416
HCV	10MYKJ019	2010	KR108538, KR108417
HCV	10MYKJ031	2010	KR108539, KR108418
HCV	10MYKJ034	2010	KR108540, KR108419
HCV	10MYKJ090	2010	KR108543, KR108420
HCV	10MYKJ093	2010	KR108544, KR108421
HCV	10MYKJ130	2010	KR108545, KR108423
HCV	10MYKJ138	2010	KR108546, KR108424
HCV	10MYKJ170	2010	KR108547, KR108425
HCV	10MYKJ208	2010	KR108549, KR108426
HCV	10MYKJ200	2010	KR108548, KR108427
HCV	10MYKJ003	2010	KR108550, KR108428
HCV	10MYKJ004	2010	KR108551, KR108429
HCV	10MYKJ015	2010	KR108552, KR108430
HCV	10MYKJ016	2010	KR108553, KR108431
HCV	10MYKJ017	2010	KR108554, KR108432
HCV	10MYKJ022	2010	KR108555, KR108433
HCV	10MYKJ027	2010	KR108556, KR108434
HCV	10MYKJ029	2010	KR108557, KR108435
HCV	10MYKJ030	2010	KR108558, KR108436
HCV	10MYKJ037	2010	KR108560, KR108437
HCV	10MYKJ046	2010	KR108562, KR108438
HCV	10MYKJ048	2010	KR108563, KR108439
HCV	10MYKJ049	2010	KR108564, KR108440
HCV	10MYKJ052	2010	KR108565, KR108441
HCV	10MYKJ054	2010	KR108566, KR108442

HCV	10MYKJ057	2010	KR108567, KR108443
HCV	10MYKJ059	2010	KR108569, KR108444
HCV	10MYKJ065	2010	KR108570, KR108446
HCV	10MYKJ066	2010	KR108571, KR108447
HCV	10MYKJ068	2010	KR108572, KR108448
HCV	10MYKJ071	2010	KR108573, KR108449
HCV	10MYKJ073	2010	KR108574, KR108450
HCV	10MYKJ074	2010	KR108575, KR108451
HCV	10MYKJ075	2010	KR108576, KR108452
HCV	10MYKJ080	2010	KR108577, KR108453
HCV	10MYKJ081	2010	KR108578, KR108454
HCV	10MYKJ084	2010	KR108579, KR108455
HCV	10MYKJ087	2010	KR108580, KR108456
HCV	10MYKJ096	2010	KR108581, KR108457
HCV	10MYKJ099	2010	KR108582, KR108458
HCV	10MYKJ102	2010	KR108583, KR108459
HCV	10MYKJ105	2010	KR108584, KR108460
HCV	10MYKJ111	2010	KR108585, KR108461
HCV	10MYKJ117	2010	KR108586, KR108462
HCV	09MYKJ128	2009	KR108587, KR108463
HCV	09MYKJ132	2009	KR108588, KR108464
HCV	09MYKJ133	2009	KR108589, KR108465
HCV	09MYKJ134	2009	KR108590, KR108466
HCV	09MYKJ140	2009	KR108591, KR108467
HCV	09MYKJ142	2009	KR108592, KR108468
HCV	09MYKJ146	2009	KR108593, KR108469
HCV	09MYKJ150	2009	KR108594, KR108470
HCV	09MYKJ152	2009	KR108595, KR108471
HCV	09MYKJ153	2009	KR108596, KR108472
HCV	09MYKJ154	2009	KR108597, KR108473
HCV	10MYKJ171	2010	KR108598, KR108474
HCV	10MYKJ175	2010	KR108599, KR108475
HCV	10MYKJ177	2010	KR108600, KR108476
HCV	10MYKJ199	2010	KR108602, KR108477
HCV	10MYKJ202	2010	KR108603, KR108478
HCV	10MYKJ203	2010	KR108604, KR108479
HCV	10MYKJ204	2010	KR108605, KR108480
HCV	10MYKJ207	2010	KR108606, KR108481
HCV	09MYKJ147	2009	KR108619, KR108483
HCV	10MYKJ011	2010	KR108608, KR108484
HCV	10MYKJ014	2010	KR108607, KR108485
HCV	10MYKJ041	2010	KR108610, KR108486
HCV	10MYKJ047	2010	KR108611, KR108487
HCV	10MYKJ086	2010	KR108612, KR108488
HCV	10MYKJ092	2010	KR108613, KR108489
HCV	10MYKJ098	2010	KR108614, KR108491
HCV	10MYKJ101	2010	KR108615, KR108492
HCV	10MYKJ108	2010	KR108616, KR108493
HCV	10MYKJ113	2010	KR108617, KR108494
HCV	09MYKJ143	2009	KR108618, KR108496
HCV	09MYKJ151	2009	KR108623, KR108497
HCV	09MYKJ137	2009	KR108411
HCV	10MYKJ116	2010	KR108495

HCV	10MYKJ097	2010	KR108490
HCV	10MYKJ209	2010	KR108482
HCV	10MYKJ060	2010	KR108445
HCV	10MYKJ104	2010	KR108422
HCV	10MYKJ005	2010	KR108499
HCV	10MYKJ007	2010	KR108500
HCV	10MYKJ023	2010	KR108504
HCV	10MYKJ036	2010	KR108505
HCV	10MYKJ077	2010	KR108514
HCV	10MYKJ107	2010	KR108519
HCV	09MYKJ120	2009	KR108522
HCV	09MYKJ121	2009	KR108523
HCV	09MYKJ135	2009	KR108526
HCV	09MYKJ145	2009	KR108528
HCV	10MYKJ178	2010	KR108531
HCV	10MYKJ210	2010	KR108533
HCV	10MYKJ212	2010	KR108534
HCV	10MYKJ213	2010	KR108535
HCV	10MYKJ218	2010	KR108536
HCV	10MYKJ079	2010	KR108541
HCV	10MYKJ082	2010	KR108542
HCV	10MYKJ033	2010	KR108559
HCV	10MYKJ040	2010	KR108561
HCV	10MYKJ058	2010	KR108568
HCV	10MYKJ179	2010	KR108601
HCV	10MYKJ114	2010	KR108607
HCV	09MYKJ143	2009	KR108618
HCV	10MYKJ201	2010	KR108620
HCV	10MYKJ219	2010	KR108621
HCV	10MYKJ032	2010	KR108622
HIV-1	09MYKJ130	2009	KR108624
HIV-1	09MYKJ136	2009	KR108625
HIV-1	09MYKJ145	2009	KR108626
HIV-1	09MYKJ146	2009	KR108627
HIV-1	10MYKJ161	2010	KR108628
HIV-1	10MYKJ165	2010	KR108629
HIV-1	10MYKJ167	2010	KR108630
HIV-1	10MYKJ170	2010	KR108631
HIV-1	10MYKJ171	2010	KR108632
HIV-1	10MYKJ174	2010	KR108633
HIV-1	10MYKJ175	2010	KR108634
HIV-1	10MYKJ182	2010	KR108635
HIV-1	10MYKJ184	2010	KR108636
HIV-1	10MYKJ192	2010	KR108637
HIV-1	10MYKJ193	2010	KR108638
HIV-1	10MYKJ195	2010	KR108639
HIV-1	10MYKJ202	2010	KR108640
HIV-1	10MYKJ203	2010	KR108641
HIV-1	10MYKJ204	2010	KR108642
HIV-1	10MYKJ208	2010	KR108643
HIV-1	10MYKJ211	2010	KR108644
HIV-1	10MYKJ001	2010	KC477938
HIV-1	10MYKJ003	2010	KC477939

HIV-1	10MYKJ005	2010	KC477941
HIV-1	10MYKJ007	2010	KC477943
HIV-1	10MYKJ009	2010	KC477944
HIV-1	10MYKJ010	2010	KC477945
HIV-1	10MYKJ011	2010	KC477946
HIV-1	10MYKJ015	2010	KC477949
HIV-1	10MYKJ016	2010	KC477950
HIV-1	10MYKJ018	2010	KC477952
HIV-1	10MYKJ020	2010	KC477953
HIV-1	10MYKJ021	2010	KC477954
HIV-1	10MYKJ022	2010	KC477955
HIV-1	10MYKJ023	2010	KC477956
HIV-1	10MYKJ025	2010	KC477957
HIV-1	10MYKJ027	2010	KC477959
HIV-1	10MYKJ028	2010	KC477960
HIV-1	10MYKJ031	2010	KC477961
HIV-1	10MYKJ032	2010	KC477962
HIV-1	10MYKJ033	2010	KC477963
HIV-1	10MYKJ036	2010	KC477966
HIV-1	10MYKJ038	2010	KC477967
HIV-1	10MYKJ042	2010	KC477970
HIV-1	10MYKJ047	2010	KC477974
HIV-1	10MYKJ049	2010	KC477975
HIV-1	10MYKJ050	2010	KC477976
HIV-1	10MYKJ051	2010	KC477977
HIV-1	10MYKJ052	2010	KC477978
HIV-1	10MYKJ053	2010	KC477979
HIV-1	10MYKJ054	2010	KC477980
HIV-1	10MYKJ055	2010	KC477981
HIV-1	10MYKJ056	2010	KC477982
HIV-1	10MYKJ057	2010	KC477983
HIV-1	10MYKJ058	2010	KC477984
HIV-1	10MYKJ059	2010	KC477985
HIV-1	10MYKJ060	2010	KC477986
HIV-1	10MYKJ061	2010	KC477987
HIV-1	10MYKJ062	2010	KC477988
HIV-1	10MYKJ063	2010	KC477989
HIV-1	10MYKJ064	2010	KC477990
HIV-1	10MYKJ065	2010	KC477991
HIV-1	10MYKJ067	2010	KC477992
HIV-1	10MYKJ069	2010	KC477994
HIV-1	10MYKJ070	2010	KC477995
HIV-1	10MYKJ072	2010	KC477996
HIV-1	10MYKJ073	2010	KC477997
HIV-1	10MYKJ074	2010	KC477998
HIV-1	10MYKJ075	2010	KC477999
HIV-1	10MYKJ076	2010	KC478000
HIV-1	10MYKJ079	2010	KC478002
HIV-1	10MYKJ081	2010	KC478003
HIV-1	10MYKJ085	2010	KC478005
HIV-1	10MYKJ086	2010	KC478006
HIV-1	10MYKJ090	2010	KC478008
HIV-1	10MYKJ092	2010	KC478009

HIV-1	10MYKJ095	2010	KC478012
HIV-1	10MYKJ096	2010	KC478013
HIV-1	10MYKJ097	2010	KC478014
HIV-1	10MYKJ099	2010	KC478016
HIV-1	10MYKJ101	2010	KC478017
HIV-1	10MYKJ104	2010	KC478019
HIV-1	10MYKJ105	2010	KC478020
HIV-1	10MYKJ107	2010	KC478021
HIV-1	10MYKJ108	2010	KC478022
HIV-1	10MYKJ109	2010	KC478023
HIV-1	10MYKJ111	2010	KC478024
HIV-1	10MYKJ112	2010	KC478025
HIV-1	10MYKJ113	2010	KC478026
HIV-1	10MYKJ114	2010	KC478027
HIV-1	10MYKJ115	2010	KC478028
HIV-1	10MYKJ117	2010	KC478030
HIV-1	10MYKJ118	2010	KC478031
HIV-1	10MYKJ119	2010	KC478032
HPgV	10MYKJ007	2010	KR108646, KR131802
HPgV	10MYKJ009	2010	KR108647, KR131795
HPgV	10MYKJ010	2010	KR108648, KR131808
HPgV	10MYKJ012	2010	KR108650, KR131793
HPgV	10MYKJ017	2010	KR108653, KR131800
HPgV	10MYKJ020	2010	KR108654, KR131812
HPgV	10MYKJ023	2010	KR108655, KR131811
HPgV	10MYKJ037	2010	KR108659, KR131792
HPgV	10MYKJ044	2010	KR108660, KR131821
HPgV	10MYKJ050	2010	KR108661, KR131809
HPgV	10MYKJ052	2010	KR108662, KR131816
HPgV	10MYKJ057	2010	KR108663, KR131801
HPgV	10MYKJ058	2010	KR108664, KR131810
HPgV	10MYKJ061	2010	KR108665, KR131796
HPgV	10MYKJ063	2010	KR108666, KR131819
HPgV	10MYKJ066	2010	KR108667, KR131818
HPgV	10MYKJ067	2010	KR108668, KR131804
HPgV	10MYKJ070	2010	KR108669, KR131788
HPgV	10MYKJ071	2010	KR108670, KR131799
HPgV	10MYKJ073	2010	KR108672, KR131822
HPgV	10MYKJ074	2010	KR108673, KR131805
HPgV	10MYKJ086	2010	KR108675, KR131794
HPgV	10MYKJ089	2010	KR108677, KR131823
HPgV	10MYKJ093	2010	KR108678, KR131814
HPgV	10MYKJ103	2010	KR108680, KR131798
HPgV	10MYKJ105	2010	KR108681, KR131806
HPgV	10MYKJ109	2010	KR108682, KR131789
HPgV	10MYKJ113	2010	KR108683, KR131797
HPgV	10MYKJ115	2010	KR108684, KR131817
HPgV	10MYKJ116	2010	KR108685, KR131790
HPgV	10MYKJ117	2010	KR108686, KR131791
HPgV	10MYKJ075	2010	KR108687, KR131824
HPgV	10MYKJ024	2010	KR108688, KR131830
HPgV	10MYKJ022	2010	KR108689, KR131831
HPgV	10MYKJ041	2010	KR108690, KR131825

HPgV	10MYKJ068	2010	KR108693, KR131826
HPgV	10MYKJ087	2010	KR108694, KR131829
HPgV	10MYKJ077	2010	KR108695, KR131827
HPgV	10MYKJ108	2010	KR108696, KR131828
HPgV	10MYKJ088	2010	KR131803
HPgV	10MYKJ085	2010	KR131807
HPgV	10MYKJ099	2010	KR131813
HPgV	10MYKJ202	2010	KR131815
HPgV	10MYKJ006	2010	KR131820
HPgV	10MYKJ056	2010	KR131832
HPgV	10MYKJ038	2010	KR131833
HPgV	10MYKJ001	2010	KR108645
HPgV	10MYKJ011	2010	KR108649
HPgV	10MYKJ013	2010	KR108651
HPgV	10MYKJ016	2010	KR108652
HPgV	10MYKJ025	2010	KR108656
HPgV	10MYKJ030	2010	KR108657
HPgV	10MYKJ032	2010	KR108658
HPgV	10MYKJ072	2010	KR108671
HPgV	10MYKJ083	2010	KR108674
HPgV	10MYKJ091	2010	KR108676
HPgV	10MYKJ094	2010	KR108679
HPgV	10MYKJ045	2010	KR108691
HPgV	10MYKJ064	2010	KR108692
